# STING-targeting PROTACs: emerging therapeutic tools for enhanced immunotherapy in inflammatory diseases

**DOI:** 10.3389/fimmu.2025.1631132

**Published:** 2025-09-15

**Authors:** Wenqing Jiang, Xiaoping Yang, Huiying Liu, Chao Wang, Hongxin Niu, Wanpeng Yu

**Affiliations:** ^1^ Qingdao Traditional Chinese Medicine Hospital, Qingdao Hiser Hospital Affiliated of Qingdao University, Qingdao, Shandong, China; ^2^ College of First Clinical Medical, Shandong University of Traditional Chinese Medicine, Jinan, China; ^3^ Qingdao Medical College, Qingdao University, Qingdao, China; ^4^ Department of Intervention, Qilu Hospital (Qingdao), Cheeloo College of Medicine, Shandong University, Qingdao, Shandong, China

**Keywords:** STING, PROTACs, immunotherapy, protein degradation, inflammatory diseases

## Abstract

The stimulator of interferon genes (STING) pathway is a central regulator of innate immunity, mediating host defense against pathogens but driving chronic inflammation when dysregulated-underpinning autoimmune diseases, fibrosis, and cancer. Traditional therapies targeting STING (e.g., small-molecule inhibitors, monoclonal antibodies) face limitations including incomplete pathway suppression, off-target effects, and reliance on continuous dosing. Proteolysis-targeting chimeras (PROTACs) offer a transformative approach by enabling selective degradation of STING, achieving sustained suppression of pathological signaling. However, critical gaps remain in understanding their selectivity for pathological vs. homeostatic STING activity, risks of immune suppression, and translational challenges. This review critically evaluates the rationale for STING degradation, with a comparative analysis of recent PROTAC designs (including warhead, E3 ligase, and linker optimization) and their pharmacokinetic/pharmacodynamic trade-offs. We address compensatory innate immune pathways, biomarker development hurdles, and safety risks, highlighting strategies to enhance specificity and clinical utility. STING-targeting PROTACs hold promise for inflammatory diseases, but their success depends on overcoming these challenges.

## 1 Introduction

Inflammatory diseases constitute a diverse group of disorders characterized by dysregulated immune responses leading to chronic tissue damage and organ dysfunction (1–3). These conditions, which include rheumatoid arthritis, inflammatory bowel diseases, systemic lupus erythematosus, and various fibrotic disorders, collectively impose a substantial burden on global healthcare systems (4–7). At the molecular level, many of these diseases share common pathogenic mechanisms involving aberrant activation of innate immune pathways, with the cyclic GMP-AMP synthase (cGAS)-STING axis emerging as a central player in disease pathogenesis (8, 9).

**Figure 1:**
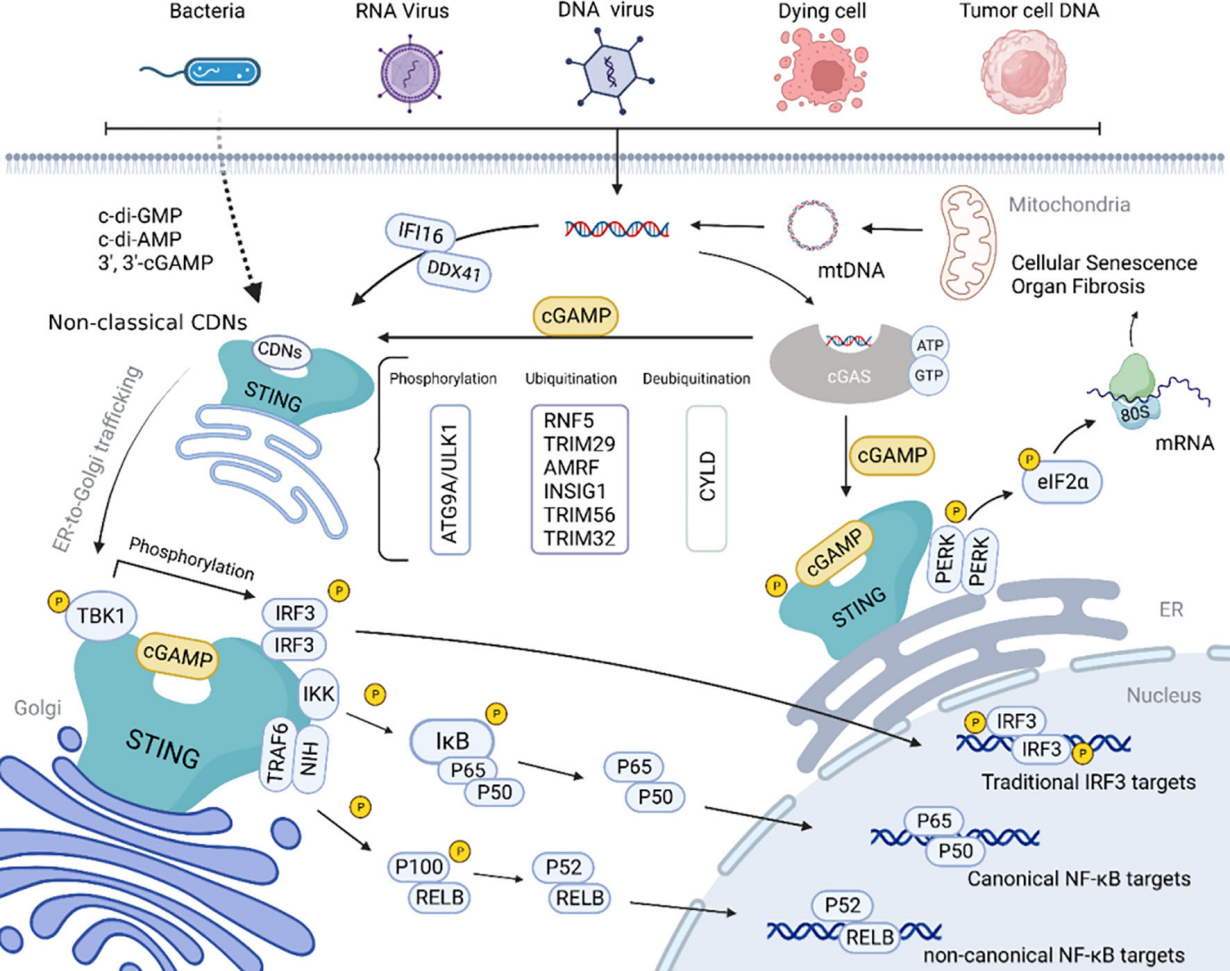
Schematic diagram of the cGAS-STING signaling pathway (10).

The cGAS-STING pathway (**Figure 1**) serves as a critical DNA sensing mechanism that detects both exogenous pathogen-derived DNA and endogenous DNA resulting from cellular damage or stress (10). Upon activation, STING initiates a cascade of downstream signaling events leading to the production of type I interferons and pro-inflammatory cytokines (11). While this response is essential for host defense against viral and bacterial infections, chronic or inappropriate activation of STING signaling has been implicated in the pathogenesis of numerous autoinflammatory and autoimmune conditions (12). For instance, gain-of-function mutations in STING are responsible for STING-associated vasculopathy with onset in infancy (SAVI), a severe autoinflammatory disorder characterized by systemic inflammation and vascular pathology (13). Similarly, excessive STING activation has been demonstrated in mouse models of lupus and rheumatoid arthritis, where it contributes to disease progression through sustained interferon production (14).

Current therapeutic strategies targeting the STING pathway in inflammatory diseases primarily focus on either inhibiting STING activation (using small molecules like C-176) or blocking downstream interferon signaling (through antibodies targeting interferon receptors or Janus kinase inhibitors) (15–17). However, these approaches suffer from several limitations: 1) incomplete pathway suppression due to the complex nature of STING signaling networks; 2) potential for compensatory activation of alternative inflammatory pathways; 3) requirement for continuous dosing due to the reversible nature of inhibition; and 4) significant off-target effects that may limit therapeutic windows (18–20).

**Figure 2:**
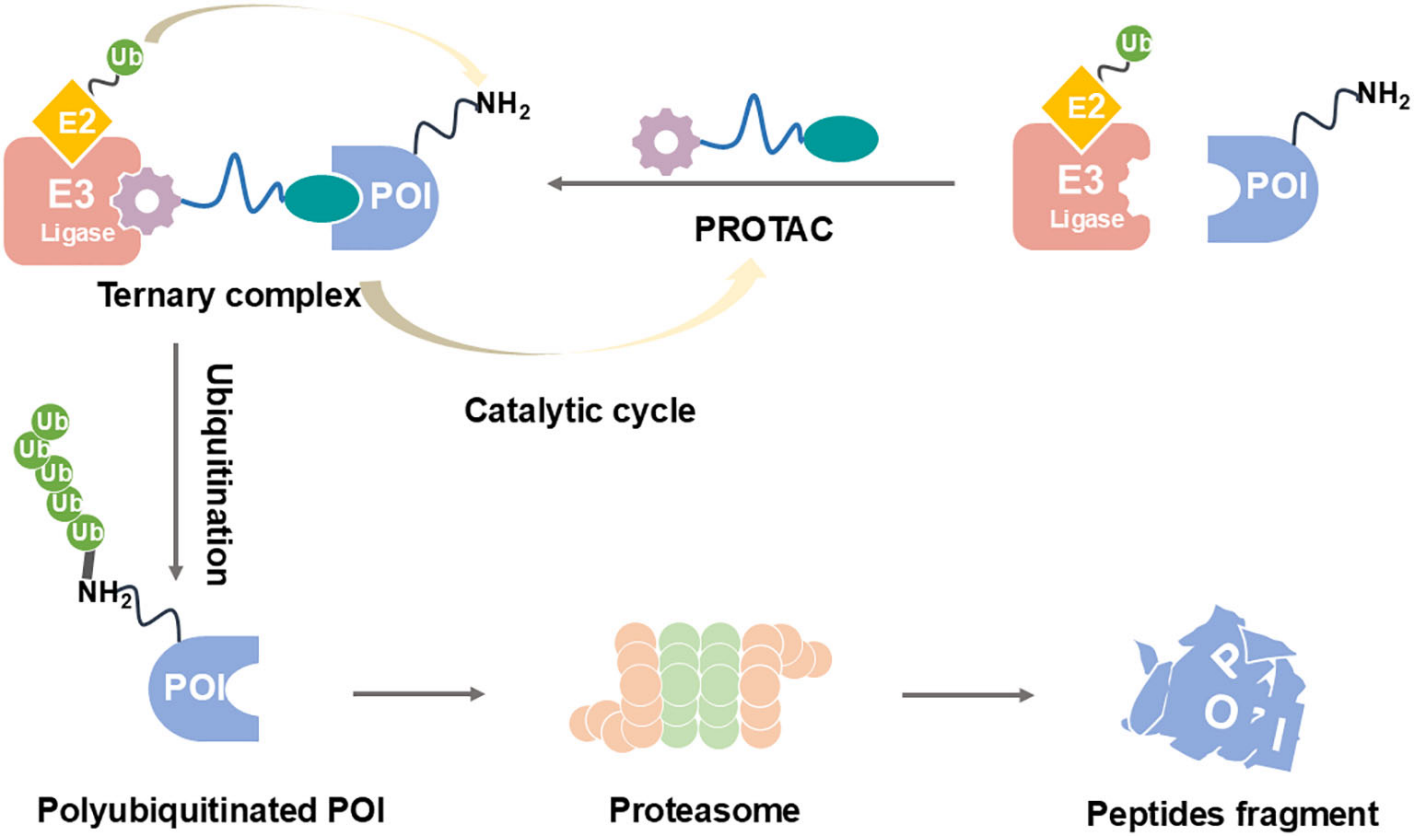
PROTAC-mediated degradation of target proteins through the UPS.

PROTAC technology (**Figure 2**) offers an innovative solution to these challenges by fundamentally changing the therapeutic approach from inhibition to elimination of the target protein (21–24). PROTACs are heterobifunctional molecules consisting of three key components: a ligand that binds the target protein (in this case, STING), a second ligand that recruits an E3 ubiquitin ligase, and a linker that connects these two moieties (25–27). This unique structure enables PROTACs to bring the target protein into proximity with an E3 ubiquitin ligase, leading to polyubiquitination and subsequent degradation by the proteasome. The catalytic nature of PROTACs allows for sustained target suppression even after the drug is cleared from the system, potentially enabling less frequent dosing regimens (28, 29). Moreover, by completely removing the target protein rather than just inhibiting its activity, PROTACs can effectively abrogate both canonical and non-canonical functions of STING, including its scaffolding roles in immune signaling complexes (30, 31).

This review will provide a comprehensive analysis of the development and potential applications of STING-targeting PROTACs in inflammatory diseases. We will begin by examining the structural and mechanistic basis for STING degradation, followed by a detailed discussion of recent advances in PROTAC design and optimization. We will then conclude with an analysis of the challenges and future directions for this promising therapeutic approach. Throughout the discussion, we will highlight how STING-directed PROTACs may overcome the limitations of current therapies and open new avenues for the treatment of refractory inflammatory conditions.

## 2 Structural and mechanistic basis for STING degradation

STING (TMEM173), a 379-amino acid transmembrane protein primarily localized to the endoplasmic reticulum (ER), has three functional domains: an N-terminal transmembrane domain (residues 1-20) anchoring it to the ER, a central cyclic dinucleotide (CDN)-binding domain (residues 139-344), and a C-terminal tail (residues 345-379) mediating downstream signaling (32, 33). Functioning as a dimer, each monomer contains a pocket binding CDNs like 2’3’-cGAMP (the endogenous cGAS product), inducing conformational changes that drive ER-to-Golgi translocation and recruitment of TBK1 and IRF3 (34, 35).

Designing effective STING-targeting PROTACs requires optimizing three components, with unique challenges posed by STING’s membrane association and structural dynamics:

- Warheads: Typically derived from STING inhibitors (e.g., amidobenzimidazoles, diABZI analogs) that compete with cGAMP for the CDN-binding pocket. Recent studies highlight the need for warheads that accommodate STING’s dimer interface to avoid partial agonism; for example, C170 analogs with modified heterocyclic rings show improved selectivity by stabilizing the inactive dimer conformation. Alternative binding sites, such as allosteric pockets near the transmembrane domain, are being explored to reduce cross-reactivity with related CDN-binding proteins.
- E3 ligase recruiters: VHL and CRBN are the most common, but their tissue distribution impacts specificity. VHL is highly expressed in immune cells (macrophages, dendritic cells), making it preferable for systemic inflammatory diseases, while CRBN’s broader expression increases risks of off-target degradation in healthy tissues (36, 37). Structural studies reveal VHL’s ligase pocket better accommodates STING’s membrane-proximal domains, explaining why VHL-recruiting PROTACs (e.g., UNC9036) exhibit higher immune cell selectivity compared to CRBN-based counterparts (e.g., SP23).
- Linkers: Critical for ternary complex formation between STING and E3 ligase, with length (5–20 atoms), flexibility, and hydrophobicity influencing efficacy. Membrane-bound STING requires linkers with sufficient hydrophobicity to traverse lipid bilayers; rigid alkyl/aromatic linkers (e.g., in ST9) improve penetration into inflamed tissues compared to flexible PEG linkers (e.g., in SP23), which enhance solubility but reduce membrane interaction (38). Computational modeling has accelerated optimization: simulations show linkers with trans-double bonds minimize steric hindrance between STING’s C-terminal tail and E3 ligases, improving degradation efficiency (39).

PROTAC-mediated STING degradation offers key advantages over inhibition: complete removal of the protein eliminates activity of constitutively active mutants (e.g., in SAVI), and catalytic activity enables sustained suppression at low doses (40, 41). Unlike inhibitors, PROTACs abrogate both canonical and non-canonical signaling, reducing interferon and cytokine production more robustly in macrophages and dendritic cells-critical for controlling dysregulated immune responses in inflammatory diseases.

## 3 Recent advances in STING-targeting PROTAC development

The development of STING-targeting PROTACs has emerged as a rapidly evolving frontier in targeted protein degradation, offering novel therapeutic avenues for both inflammatory disorders and oncological indications. Over the past several years, this field has witnessed remarkable progress, transitioning from initial proof-of-concept studies to sophisticated drug development campaigns with increasing clinical relevance. The strategic exploitation of the ubiquitin-proteasome system to modulate STING signaling represents a paradigm shift in pharmacological intervention, combining the precision of targeted therapy with the advantages of complete protein removal.

The seminal work by Liu et al. (2022) marked a watershed moment in this field through their development of SP23 (**Table 1**), the first-in-class STING-targeting PROTAC (42). This innovative molecule ingeniously combined the STING inhibitor C-170 with the cereblon E3 ligase ligand pomalidomide, creating a bifunctional degrader with impressive biochemical properties. Demonstrating a DC_50_ of 3.2 μM in cellular assays, SP23 not only effectively degraded STING but also potently suppressed cGAMP-induced inflammatory signaling in THP-1 monocytes. The therapeutic potential of this approach was further validated in preclinical models of cisplatin-induced nephrotoxicity, where SP23 outperformed its parent compound C-170 in mitigating renal inflammation and injury, thereby establishing PROTAC technology as a viable strategy for STING pathway modulation.

**Table 1A:** Representative STING-targeting degraders.

Compounds	Target protein	Structure	Ref.
SP23	STING	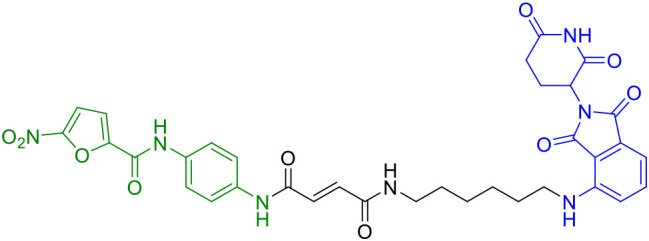	(42)
CN0	PARP1 cGAS/STING	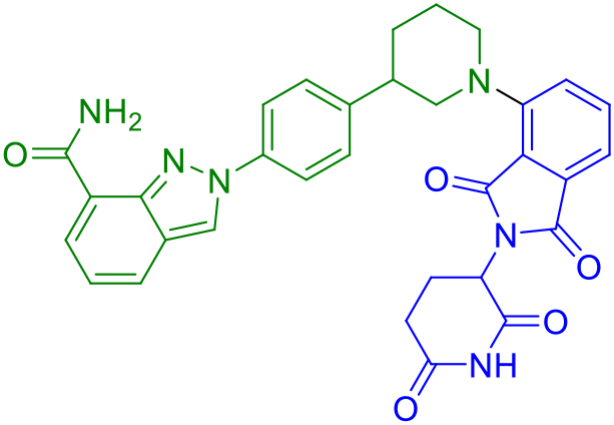	(43)
UNC9036	STING	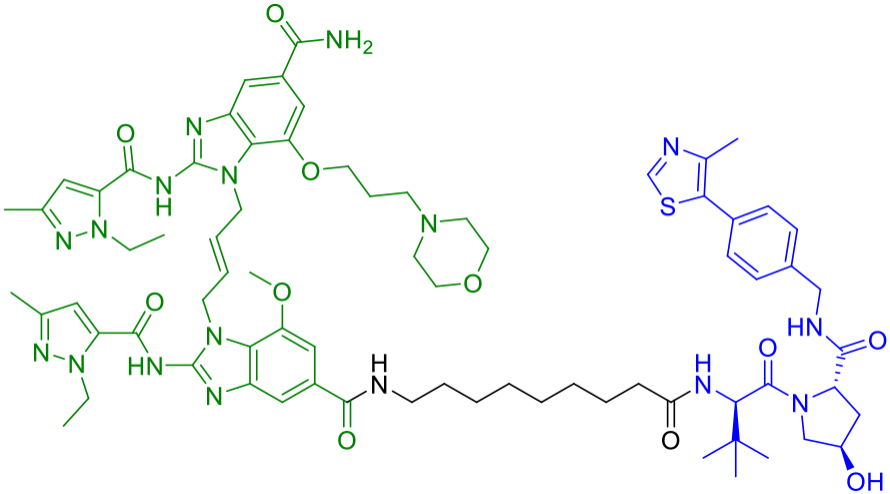	(40)
CDTAC	PD-L1/STING	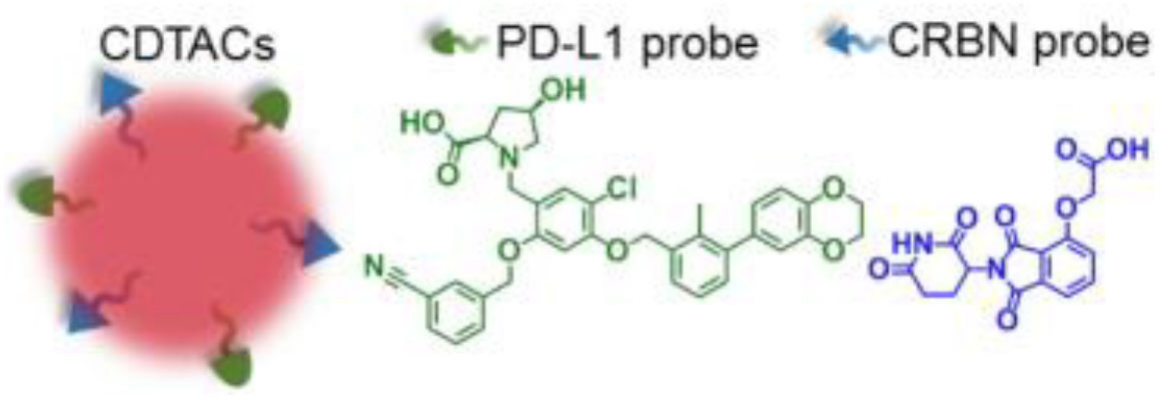	(44)
dBET6	BET/STING	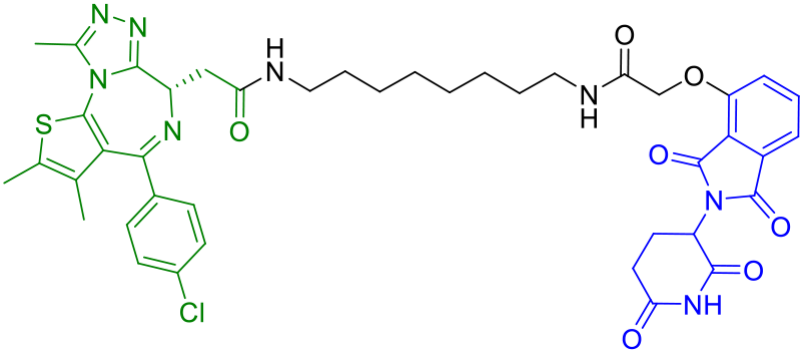	45
SD02	STING	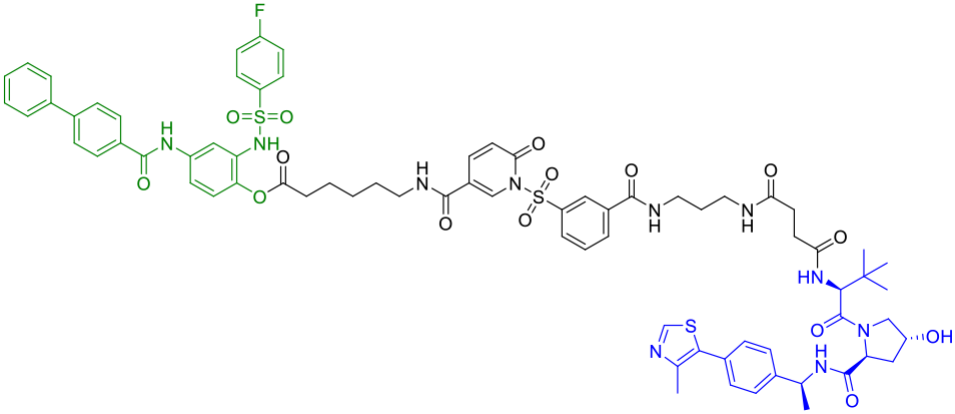	(41)
AK59	STING	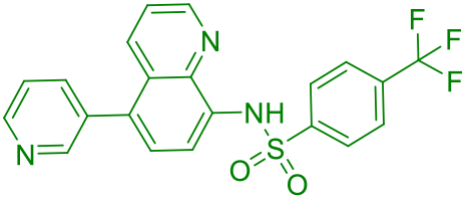	(46)
P8	STING	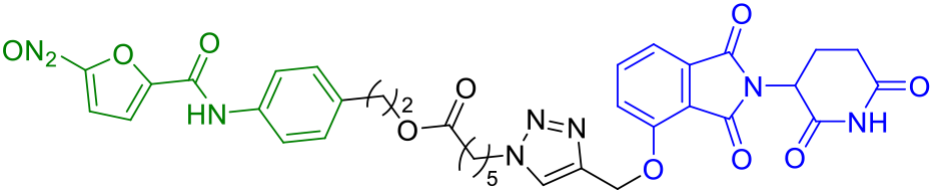	(47)
Degrader 2	STING	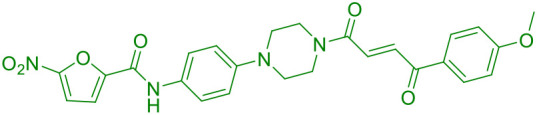	(48)
ST9	STING	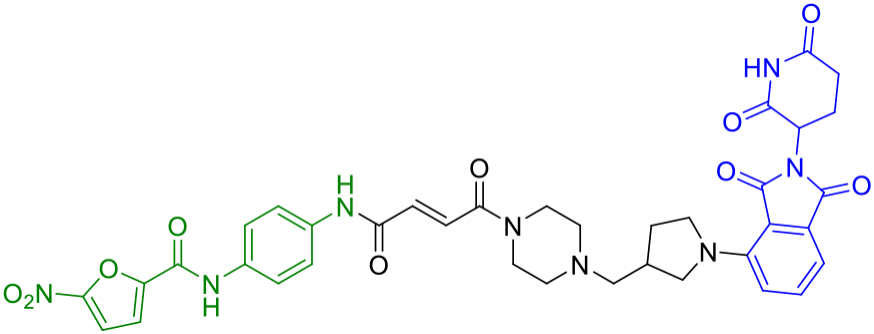	(49)
TH35	cGAS-STING	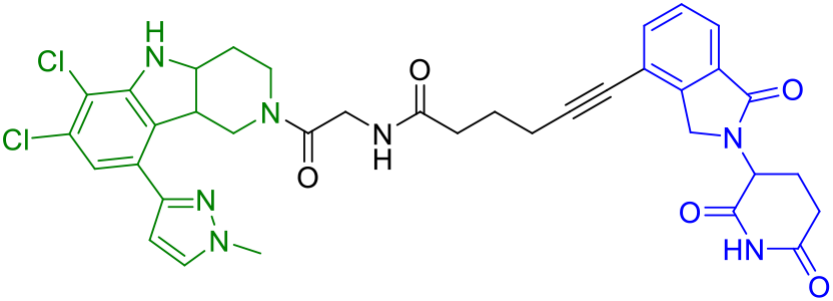	(50)
2h	STING	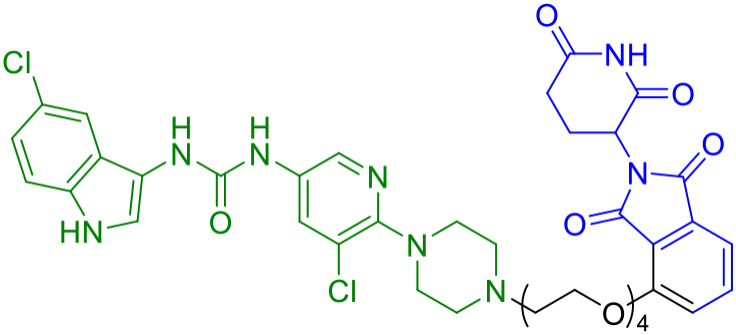	(51)

**Table 1B:** Key STING-targeting PROTACs and their properties.

Compound	DC_50_	E3 ligase	Linker type	Key advantage	Limitation
SP23	3.2 μM	CRBN	Flexible PEG	First-in-class; *in vivo* efficacy	Poor membrane penetration
UNC9036	1.8 μM	VHL	Rigid alkyl	Immune cell selectivity	Short half-life (2.3 h)
SD02	0.53 μM	CRBN	Covalent sulfonyl	Sustained degradation	Off-target cysteine modification
ST9	0.62 μM	VHL	Rigid aromatic	Renoprotective efficacy	Poor BBB penetration
TH35	2.1 μM	CRBN	Mixed PEG-alkyl	Dual cGAS-STING targeting	cGAS cross-degradation

In the same year, Lin et al. developed a novel series of PROTACs specifically designed to target and degrade PARP1 protein. Their research revealed that CN0 (**Table 1**), which lacks a PEG linker, effectively induces PARP1 degradation *via* the proteasome-mediated pathway (43). Significantly, CN0 was found to disrupt DNA damage repair mechanisms. When combined with daunorubicin (DNR), this compound led to substantial accumulation of cytosolic DNA fragments due to persistent unrepaired DNA lesions. This biological effect subsequently triggered activation of the cGAS/STING (cyclic GMP-AMP synthase/stimulator of interferon genes) pathway, a crucial component of innate immunity. The resulting inflammatory signaling cascade contributed to tumor microenvironment remodeling, potentially enhancing the cytotoxic T cell response against tumor cells.

Subsequent research efforts have significantly expanded the mechanistic diversity and therapeutic scope of STING PROTACs. Zhu et al. (2023) introduced a conceptually innovative approach by developing agonist-derived PROTACs capable of both activating and degrading phosphorylated STING in renal carcinoma models (40). Their work provided fundamental insights into STING regulation by identifying VHL as an endogenous E3 ligase involved in STING turnover, revealing how PROTAC (UNC9036, **Table 1**) can co-opt natural protein quality control mechanisms. In a parallel development, Su et al. (2023) broke new ground by creating carbon-dot-based PROTAC conjugates (CDTAC, **Table 1**) that uniquely combined membrane protein degradation (notably PD-L1) with STING pathway activation (44). This nanotechnology approach demonstrated enhanced tumor targeting capabilities and synergistic immunostimulatory effects when combined with metabolic modulation through fasting-mimicking diets.

In 2023, Zhu et al. demonstrated that dBET6 (**Table 1**), a PROTAC molecule targeting BET proteins for proteasomal degradation, exerts neuroprotective effects in light-induced retinal degeneration (45). Their study showed that intraperitoneal administration of dBET6 effectively preserved retinal function and structure while suppressing light damage-induced microglial activation and photoreceptor apoptosis. Single-cell RNA sequencing revealed that dBET6 specifically inhibited the cGAS-STING pathway in retinal microglia, attenuating neuroinflammation and suggesting its therapeutic potential for retinal degenerative diseases. The researchers established that light exposure activated the cGAS-STING inflammatory cascade in retinal microglia, while dBET6 treatment significantly reduced STING expression and associated inflammatory responses. These findings position targeted BET protein degradation as a promising strategy for treating retinal degeneration by modulating neuroimmune interactions through the cGAS-STING pathway.

The year 2024 represented a period of substantial technological advancement in STING PROTAC development, with multiple groups reporting significant improvements in compound design and biological activity. Luo et al. made notable progress with SD02, (**Table 1**), a covalent STING PROTAC featuring a sulfonylpyridone warhead that demonstrated exquisite selectivity for tyrosine and lysine residues (41). With its sub-micromolar DC_50_ (0.53 μM) and improved safety profile, SD02 represented a quantum leap over first-generation degraders. Simultaneously, Mutlu et al. employed comprehensive screening approaches to identify AK59 (**Table 1**), a compound that promoted site-specific ubiquitination of STING at K150 through HERC4 recruitment, showing particular efficacy against gain-of-function STING mutants associated with autoinflammatory diseases (46). Another significant contribution came from the development of P8, a CRBN-based PROTAC that exhibited fascinating cell-type dependent behavior - functioning as a degrader in THP-1 cells while paradoxically acting as an inhibitor in RAW264.7 macrophages, underscoring the complex contextual biology of PROTAC activity (47).

Recent breakthroughs have further refined the molecular architecture and therapeutic potential of STING PROTACs. Nakamura et al. (2024) revolutionized PROTAC design by developing minimal covalent handle technology, creating compact degraders (<700 Da) with sustained micromolar activity and improved bioavailability (48). These optimized constructs demonstrated enhanced selectivity for STING and superior pharmacokinetics, enabling prolonged pathway modulation ideal for chronic autoinflammatory diseases. In preclinical models of lupus and SAVI, the representative PROTAC (Degrader 2, **Table 1**) achieved durable suppression of interferon responses with less frequent dosing, marking a significant advance in targeted protein degradation therapeutics. The most recent advancement by Ma et al. (2025) represents the current state-of-the-art, with their development of ST9 (**Table 1**) featuring optimized rigid linkers that achieved a DC_50_ of 0.62 μM while significantly improving drug-like properties (49). Through systematic structure-activity relationship studies, the team elucidated critical design principles, particularly the importance of 4’-position linking and trans-double bond conformation. ST9 demonstrated exceptional renoprotective efficacy in acute kidney injury models through precise modulation of the STING/TBK1/NF-κB signaling cascade.

He et al. recently developed a novel series of CRBN-recruiting PROTAC degraders targeting cGAS-STING (**Table 1**), conducting comprehensive studies on their design, synthesis, structure-activity relationships, and biological evaluation (50). Their research identified TH35 as the most promising candidate, demonstrating potent and selective cGAS-STING degradation capability. This compound effectively suppressed dsDNA-triggered activation of cGAS-STING signaling pathways in both human and murine cell lines while showing negligible cytotoxicity. Notably, *in vivo* studies revealed that TH35 exhibited enhanced therapeutic effects compared to conventional cGAS-STING inhibitors in a DSS-induced ulcerative colitis murine model. The compound also presented favorable pharmacokinetic characteristics. As the pioneering CRBN-based cGAS-STING-targeting PROTAC degrader, TH35 represents a significant advancement in modulating anti-inflammatory responses and provides a novel therapeutic strategy for managing cGAS-STING-mediated inflammatory disorders.

Subsequently, An et al. developed novel STING-PROTACs (**Table 1**) using a non-nitrated covalent warhead coupled with various E3 ligase ligands (51). Their lead compound, 2h, demonstrated selective STING protein degradation via the proteasomal pathway, achieving a DC_50_ of 3.23 μM while maintaining sustained degradation efficacy for 72 hours. Comprehensive biological evaluation revealed that 2h effectively suppressed STING-dependent signaling pathways and significantly reduced pro-inflammatory cytokine production across cellular and animal models. Importantly, this compound exhibited an improved safety profile compared to both its parent warhead and the reference compound SP23, positioning it as a promising nitro-free covalent STING degrader for further therapeutic development.

The collective progress in STING-targeting PROTACs reflects the maturation of this therapeutic approach from exploratory research to preclinical development. Current innovations span multiple dimensions, including novel E3 ligase engagement strategies, advanced covalent targeting methodologies, and structure-guided molecular optimization. These developments have not only deepened our understanding of STING biology and regulation but have also created a robust pipeline of therapeutic candidates with potential applications across a spectrum of diseases, from autoimmune disorders to cancer immunotherapy. As the field continues to evolve, the integration of PROTAC technology with other therapeutic modalities and delivery systems promises to further expand the clinical potential of STING modulation, positioning this approach at the forefront of next-generation protein degradation therapeutics.

## 4 Challenges and future perspectives

The development of STING-targeting PROTACs presents several critical challenges that must be addressed to ensure their clinical success. These challenges span selectivity, biomarker development, and delivery optimization, each requiring innovative solutions to balance efficacy with safety.

### 4.1 Selectivity vs. immune suppression

A major concern with STING-targeting PROTACs is their potential to disrupt physiological immune defenses. While preclinical studies demonstrate preserved antiviral responses in acute settings, chronic STING degradation could impair tumor surveillance or increase susceptibility to infections. For instance, complete STING ablation might compromise the body’s ability to detect cytosolic DNA from pathogens or malignant cells, leading to unintended immunosuppression. To mitigate these risks, researchers are exploring advanced PROTAC designs. Conditionally active PROTACs, which utilize stimuli-responsive linkers (e.g., ROS- or pH-sensitive), could restrict activity to inflammatory microenvironments, sparing homeostatic STING functions. Additionally, dual-target degraders that simultaneously inhibit compensatory pathways (e.g., TLRs or RLRs) may prevent immune escape and maintain broader immune surveillance. However, these strategies require rigorous validation to ensure they do not introduce new off-target effects or complicate pharmacokinetics.

### 4.2 Biomarker development

The translational potential of STING PROTACs hinges on reliable biomarkers for patient stratification and treatment monitoring. Current efforts face significant hurdles. STING expression varies widely across tissues, complicating the identification of universal biomarkers. For example, STING levels in monocytes may not correlate with activity in epithelial cells or fibroblasts. Additionally, detecting cGAMP, the key metabolite of STING activation, remains technically challenging due to its low abundance and rapid turnover. Emerging ultrasensitive assays, such as mass spectrometry-based methods, show promise but lack standardization. Alternatively, interferon-stimulated gene (ISG) signatures, particularly a 3-gene panel (IFI27, IFI44L, SIGLEC1), offer a surrogate measure of pathway activity. However, their clinical utility depends on validation in diverse patient cohorts and disease contexts. Without robust biomarkers, dosing and efficacy assessments will remain empirical, limiting personalized therapeutic approaches.

### 4.3 Delivery and safety

PROTACs face inherent pharmacokinetic challenges due to their intermediate molecular weight (800–1200 Da), which limits oral bioavailability and tissue penetration. To overcome this, novel formulations are under investigation, including nanoparticle encapsulation to enhance stability and targeted delivery. Inhalable dry powders, for instance, could localize PROTACs to lung tissues in pulmonary inflammatory diseases, minimizing systemic exposure. Gene therapy approaches, such as AAV-mediated PROTAC delivery, offer the potential for sustained STING degradation with a single dose. However, viral vectors pose immunogenicity risks, particularly in repeat-dosing scenarios, and may be inefficient in fibrotic or inflamed tissues. Safety profiling is another critical gap. While PROTACs’ catalytic mechanism reduces the need for high doses, their prolonged action raises concerns about unintended protein degradation or immune dysfunction. Comprehensive proteome-wide analyses are needed to identify off-target effects, and chronic toxicity studies must evaluate the consequences of sustained STING suppression. Furthermore, the impact of PROTACs on immune cell subsets (e.g., dendritic cells, macrophages) requires detailed characterization to avoid disrupting immune homeostasis.

### 4.4 Future directions

Addressing these challenges will require interdisciplinary collaboration. Advances in linker chemistry, such as photoactivatable or enzyme-cleavable designs, could improve spatiotemporal control of PROTAC activity. Computational modeling and high-throughput screening may identify optimal E3 ligase-warhead pairs for tissue-specific degradation. Clinically, phase 0 trials with biomarker-driven endpoints could accelerate translational progress. By integrating these innovations, STING-targeting PROTACs may achieve the precision needed to revolutionize inflammatory disease therapy while minimizing systemic risks.

## 5 Conclusions

STING-targeting PROTACs represent a paradigm shift in the therapeutic approach to inflammatory diseases, moving beyond traditional protein inhibition to achieve complete pathway ablation through targeted degradation. This innovative strategy addresses several critical limitations of current therapies by offering (1): catalytic, long-lasting effects that enable less frequent dosing (2); elimination of both enzymatic and scaffolding functions of STING; and (3) broad potential applicability across autoimmune disorders, fibrotic conditions, and neurodegenerative diseases. The field has witnessed remarkable progress in recent years, with multiple PROTAC candidates now advancing through IND-enabling studies, suggesting that first-in-human trials may commence within the next 2–3 years.

The clinical translation of these agents will require careful consideration of several key factors. First, patient stratification strategies must be developed to identify individuals most likely to benefit from STING degradation, potentially through biomarkers such as STING expression levels or interferon signatures. Second, delivery systems need to be optimized for specific disease indications - for instance, inhalable formulations for pulmonary inflammation versus systemic delivery for autoimmune conditions. Most crucially, comprehensive safety monitoring protocols must be implemented to assess potential immunosuppressive effects and off-target degradation events, particularly given the essential role of STING in host defense mechanisms.

From a broader perspective, the development of STING-targeting PROTACs has important implications for immunology and drug discovery. These molecules serve as proof-of-concept for targeting traditionally “undruggable” membrane-associated proteins, expanding the potential applications of protein degradation technology. The growing understanding of E3 ligase biology and ubiquitin-proteasome system dynamics continues to inform the design of increasingly sophisticated degraders. Current research efforts are particularly focused on (1): developing tissue-specific delivery platforms (2); creating conditionally active PROTACs that respond to inflammatory stimuli; and (3) identifying robust biomarkers for patient selection and treatment monitoring.

Looking forward, while significant challenges remain in clinical implementation, the preclinical data generated to date provide strong justification for cautious optimism. The coming years will be critical for determining whether the theoretical advantages of STING PROTACs can be translated into meaningful clinical benefits. Key questions that remain to be answered include (1): the durability of therapeutic effects in chronic inflammatory conditions (2); the potential for compensatory activation of alternative immune pathways; and (3) the long-term safety profile of sustained STING degradation. As these molecules progress through clinical development, they will not only test the viability of PROTAC technology for inflammatory diseases but also provide valuable insights into the fundamental biology of STING signaling and immune regulation.

The first generation of clinical trials will be particularly informative, serving as a crucial test of whether this innovative approach can overcome the limitations of current therapies while maintaining an acceptable safety profile. Regardless of the ultimate clinical outcomes, the development of STING-targeting PROTACs has already contributed significantly to our understanding of targeted protein degradation and opened new avenues for therapeutic intervention in inflammatory diseases. With continued refinement and clinical validation, these molecules may eventually offer new hope for patients with refractory inflammatory conditions that are poorly served by existing treatment options.
